# Supplementary material for: The development and validation of a survey to measure fecal-oral child exposure to zoonotic enteropathogens: The FECEZ Enteropathogens Index
Source: PLOS Glob Public Health. 2024 Sep 10;4(9):e0002690. doi: 10.1371/journal.pgph.0002690 (PMC11386431; doi:10.1371/journal.pgph.0002690)

The development and validation of a survey to measure fecal-oral child exposure to zoonotic enteropathogens: The FECEZ Enteropathogens Index

S3 Fig

April M. Ballard[a,b], Regine Haardörfer[c], Betty Corozo Angulo[d], Matthew C. Freeman[b], Joseph N.S. Eisenberg[e], Gwenyth O. Lee[f], Karen Levy[g], Bethany A. Caruso[h]

[a] Department of Population Health Sciences, Georgia State University School of Public Health

[b] Gangarosa Department of Environmental Health, Emory University Rollins School of Public Health

[c] Department of Behavioral, Social, and Health Education Sciences, Emory University Rollins School of Public Health

[d] Universidad Técnica Luis Vargas Torres de Esmeraldas

[e] Department of Epidemiology, University of Michigan School of Public Health

[f] Rutgers Global Health Institute and Department of Biostatistics and Epidemiology, Rutgers School of Public Health

[g] Department of Environmental and Occupational Health Sciences, University of Washington School of Public Health

[h] Hubert Department of Global Health, Emory University Rollins School of Public Health

S3 Fig. Histogram of Child *Behavior* sub-domain scores (possible score range: 0-42, *n*=297)

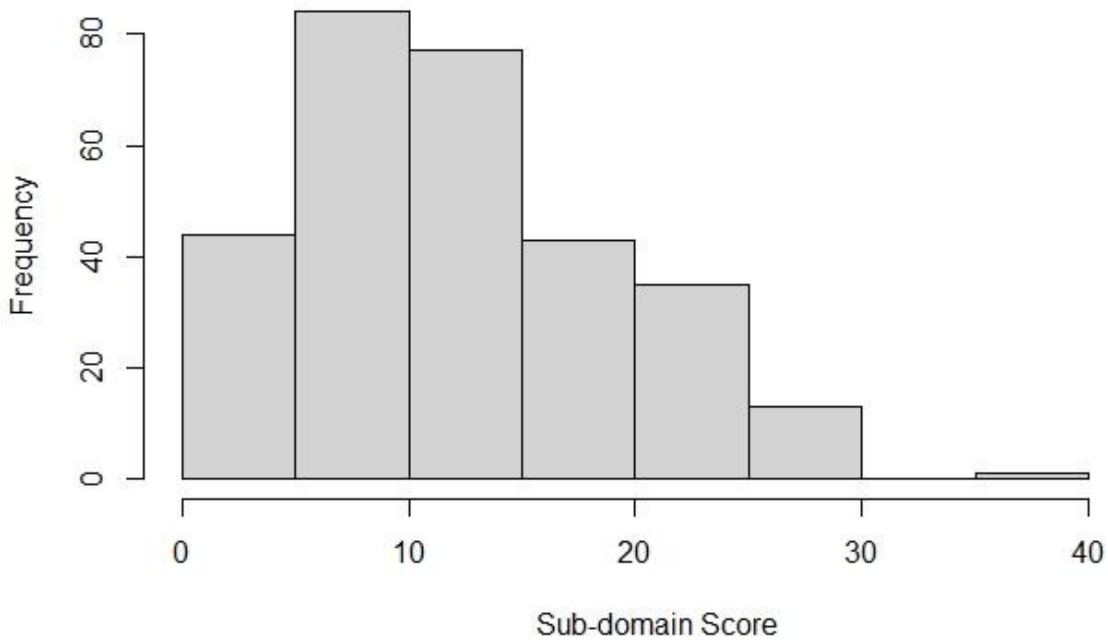

Supplement: S3 Fig — (PDF) [file pgph.0002690.s004.pdf]
