# Supplementary material for: The development and validation of a survey to measure fecal-oral child exposure to zoonotic enteropathogens: The FECEZ Enteropathogens Index
Source: PLOS Glob Public Health. 2024 Sep 10;4(9):e0002690. doi: 10.1371/journal.pgph.0002690 (PMC11386431; doi:10.1371/journal.pgph.0002690)
Supplement: S1 Table — (PDF) [file pgph.0002690.s006.pdf]

The development and validation of a survey to measure fecal-oral child exposure to zoonotic enteropathogens: The FECEZ Enteropathogens Index

S1 Table

April M. Ballard[a,b], Regine Haardörfer[c], Betty Corozo Angulo[d], Matthew C. Freeman[b], Joseph N.S. Eisenberg[e], Gwenyth O. Lee[f], Karen Levy[g], Bethany A. Caruso[h]

[a] Department of Population Health Sciences, Georgia State University School of Public Health

[b] Gangarosa Department of Environmental Health, Emory University Rollins School of Public Health

[c] Department of Behavioral, Social, and Health Education Sciences, Emory University Rollins School of Public Health

[d] Universidad Técnica Luis Vargas Torres de Esmeraldas

[e] Department of Epidemiology, University of Michigan School of Public Health

[f] Rutgers Global Health Institute and Department of Biostatistics and Epidemiology, Rutgers School of Public Health

[g] Department of Environmental and Occupational Health Sciences, University of Washington School of Public Health

[h] Hubert Department of Global Health, Emory University Rollins School of Public Health

S1 Table. Table of survey items grouped by sub-domain and post-PCA results

Item #	Sub-domain and associated survey items	PCA result	Reason for dropping
	Child Environment (CE)		
CE1	Production chickens spend time outside near the house	Dropped	All responded “never”
CE2	Production chickens enter the house	Dropped	All responded “never”
CE3	Production chickens sleep inside the house	Dropped	All responded “never”
CE4	Ducks spend time outside near the house	Dropped	NZP, not relevant for this sample
CE5	Ducks enter the house	Dropped	NZP, not relevant for this sample
CE6	Ducks sleep inside the house	Dropped	All participants responded “never”
CE7	Turkeys spend time outside near the house	Dropped	All participants responded “never”
CE8	Turkeys enter the house	Dropped	All participants responded “never”
CE9	Turkeys sleep inside the house	Dropped	All participants responded “never”
CE10	Guinea pigs spend time outside near the house	Dropped	All participants responded “never”
CE11	Guinea pigs enter the house	Dropped	All participants responded “never”
CE12	Guinea pigs sleep inside the house	Dropped	All participants responded “never”
CE13	Dogs spend time outside near the house	CE dog principal component (PC)	--
CE14	Dogs enter the house	CE maternal PC	--
CE15	Dogs sleep inside the house	CE dog PC	--
CE16	Pigs outside the house near or in the yard	Dropped	NZP, all loadings <|0.4|

Item #	Sub-domain and associated survey items	PCA result	Reason for dropping
	Child Environment (CE)		
CE17	Pigs enter the house	Dropped	NZP, not relevant for this sample
CE18	Pigs sleep inside the house	Dropped	NZP, not relevant for this sample
CE19	Cattle spend time outside near the house	Dropped	All participants responded “never”
CE20	Cattle enter the house	Dropped	All participants responded “never”
CE21	Cattle sleep inside the house	Dropped	All participants responded “never”
CE22	Dairy cattle spend time outside near the house	CE dairy cattle PC	--
CE23	Dairy cattle enter the house	Dropped	All participants responded “never”
CE24	Dairy cattle sleep in the house	Dropped	All participants responded “never”
CE25	Horses/mules/donkeys spend time outside near the house	Dropped	NZP, not relevant for this sample
CE26	Horses/mules/donkeys enter the house	Dropped	All participants responded “never”
CE27	Horses/mules/donkeys sleep inside the house	Dropped	All participants responded “never”
CE28	Sheep/goats spend time outside near the house	Dropped	NZP, not relevant for this sample
CE29	Sheep/goats enter the house	Dropped	NZP, not relevant for this sample
CE30	Sheep/goats sleep inside the house	Dropped	NZP, not relevant for this sample
CE31	Cats spend time outside near the house	CE cat PC	--
CE32	Cats enter the house	CE cat PC	--
CE33	Cats sleep inside the house	CE cat PC	--
CE34	Creole chickens sleep inside the house	Dropped	NZP, all loadings <|0.4|
CE35	Creole chickens enter the house	CE creole chicken PC	--

Item #	Sub-domain and associated survey items	PCA result	Reason for dropping
	Child Environment (CE)		
CE36	Creole chickens sleep inside the house	CE creole chicken PC	--
CE37	Bushrats spend time outside near the house	Dropped	All participants responded “never”
CE38	Bushrats enter the house	Dropped	All participants responded “never”
CE39	Bushrats sleep inside the house	Dropped	All participants responded “never”
CE40	Rats/rodents spend time outside near the house	Dropped	NZP, not relevant for this sample
CE41	Rats/rodents enter the house	Dropped	NZP, not relevant for this sample
CE42	Rats/rodents sleep inside the house	Dropped	NZP, not relevant for this sample
CE43	Rabbits spend time outside near the house	Dropped	NZP, not relevant for this sample
CE44	Rabbits enter the house	Dropped	NZP, not relevant for this sample
CE45	Rabbits sleep inside the house	Dropped	NZP, not relevant for this sample
CE46	Production chicken poop outside the house near or in the yard	Dropped	All participants responded “never”
CE47	Production chicken poop inside the house	Dropped	All participants responded “never”
CE48	Duck poop outside the house near or in the yard	Dropped	NZP, not relevant for this sample
CE49	Duck poop inside the house	Dropped	NZP, not relevant for this sample
CE50	Turkey poop outside the house near or in the yard	Dropped	All participants responded “never”
CE51	Turkey poop inside the house	Dropped	All participants responded “never”
CE52	Guinea pig poop outside the house near or in the yard	Dropped	All participants responded “never”
CE53	Guinea pig poop inside the house	Dropped	All participants responded “never”

Item #	Sub-domain and associated survey items	PCA result	Reason for dropping
	Child Environment (CE)		
CE54	Dog poop outside the house near or in the yard	CE dog PC	--
CE55	Dog poop inside the house	Dropped	NZP, all loadings <|0.4|
CE56	Pig poop outside the house near or in the yard	Dropped	NZP, not relevant for this sample
CE57	Pig poop inside the house	Dropped	All participants responded “never”
CE58	Cattle poop outside the house near or in the yard	Dropped	All participants responded “never”
CE59	Cattle poop inside the house	Dropped	All participants responded “never”
CE60	Dairy cattle poop outside the house near or in the yard	CE dairy cattle PC	--
CE61	Dairy cattle poop inside the house	Dropped	All participants responded “never”
CE62	Horses/mule/donkey poop outside the house near or in the yard	Dropped	NZP, all loadings <|0.4|
CE63	Horses/mule/donkey poop inside the house	Dropped	All participants responded “never”
CE64	Sheep/goat poop outside the house near or in the yard	Dropped	All participants responded “never”
CE65	Sheep/goat poop inside the house	Dropped	All participants responded “never”
CE66	Cat poop outside the house near or in the yard	CE cat PC	--
CE67	Cat poop inside the house	Dropped	NZP, all loadings <|0.4|
CE68	Creole chicken poop outside the house near or in the yard	CE creole chicken PC	--
CE69	Creole chicken poop inside the house	CE creole chicken PC	--
CE70	Bushrat poop outside the house near or in the yard	Dropped	All participants responded “never”

Item #	Sub-domain and associated survey items	PCA result	Reason for dropping
	Child Environment (CE)		
CE71	Bushrat poop inside the house	Dropped	All participants responded “never”
CE72	Rat/rodent poop outside the house near or in the yard	Dropped	NZP, not relevant for this sample
CE73	Rat/rodent poop inside the house	Dropped	NZP, not relevant for this sample
CE74	Rabbit poop outside the house near or in the yard	Dropped	NZP, not relevant for this sample
CE75	Rabbit poop inside the house	Dropped	NZP, not relevant for this sample
CE76	Poop from an unknown type of animal outside the house near or in the yard	Dropped	All participants responded “never”
CE77	Poop from an unknown type of animal inside the house	Dropped	All participants responded “never”
CE90	House member (apart from mother and child under 5 years) work or care for an animal	CE dairy cattle PC	--
CE91	Mother or someone who lives with you put or throw leftover food outside for an animal	Dropped	All loadings <|0.4|
CE92	Mother personally feeds or gives water to an animal	CE maternal PC	--
CE93	Mother personally touches or plays with an animal	CE maternal PC	--
CE94	Mother personally bathes, cleans, or grooms an animal	CE maternal PC	--
CE95	Mother personally cleans the habitat or place where an animal sleeps and/or defecates	CE maternal PC	--
CE96	Mother personally cares for an animal that was sick	Dropped	NZP, all loadings <|0.4|
CE97	Mother personally eliminates or cleans the poop of an animal	CE maternal PC	--

Item #	Sub-domain and associated survey items	PCA result	Reason for dropping
	Child Behavior (CB)		
CB78	Child plays on the floor of the house without a rug or playmat	Dropped	All loadings <|0.4|
CB79	Child plays inside the house in an area where an animal spends time or sleeps	Dropped	All loadings <|0.4|
CB80	Child plays with or carries around shoes like a toy	CB play and mouthing PC	
CB81	Child plays in soil or dirt outside the house	CB play and mouthing PC	--
CB82	Child play in sand outside the house	Dropped	Consolidated with CB81 due to high correlations (p>0.9)
CB83	Child plays outside the house in an area where an animal lives or sleeps	CB play and mouthing PC	--
CB84	Child plays outside the house without shoes on	CB play and mouthing PC	--
CB85	Child puts objects or toys that had contact with the floor inside the house in their mouth	CB play and mouthing PC	--
CB86	Child puts objects or toys that had contact with the dirt outside the house in their mouth	CB play and mouthing PC	--
CB87	Child puts dirt or soil in their mouth	CB play and mouthing PC	--
CB88	Child put sand in their mouth	Dropped	Consolidated with CB87 due to high correlations (p>0.9)
CB89	Child puts shoes in their mouth	CB play and mouthing PC	--
CB98	Child feeds or gives water or helps others feed or give water to an animal	CB animal caregiving and feces management PC	--

Item #	Sub-domain and associated survey items	PCA result	Reason for dropping
	Child Behavior (CB)		
CB99	Child touches or plays with an animal	CB animal caregiving and feces management PC	--
CB100	Child bathes, cleans, or grooms or helps others bathe, clean, or groom an animal	CB animal caregiving and feces management PC	--
CB101	Child cleans or helps others clean the habitat or place where an animal sleeps and/or defecates	CB animal caregiving and feces management PC	--
CB102	Child cares for or helps others care for an animal that was sick	CB animal caregiving and feces management PC	--
CB103	Child touches or plays with objects used to remove or clean animal poop such as brooms or shovels	Dropped	NZP, all loadings <|0.4|
CB104	Child touches, removes, or cleans animal poop	CB animal caregiving and feces management PC	--
CB105	Child put animal poop in their mouth	Dropped	All participants responded "never"
