# Supplementary material for: The development and validation of a survey to measure fecal-oral child exposure to zoonotic enteropathogens: The FECEZ Enteropathogens Index
Source: PLOS Glob Public Health. 2024 Sep 10;4(9):e0002690. doi: 10.1371/journal.pgph.0002690 (PMC11386431; doi:10.1371/journal.pgph.0002690)
Supplement: S2 Table — (PDF) [file pgph.0002690.s007.pdf]

The development and validation of a survey to measure fecal-oral child exposure to zoonotic enteropathogens: The FECEZ Enteropathogens Index

S2 Table

April M. Ballard[a,b], Regine Haardörfer[c], Betty Corozo Angulo[d], Matthew C. Freeman[b], Joseph N.S. Eisenberg[e], Gwenyth O. Lee[f], Karen Levy[g], Bethany A. Caruso[h]

[a] Department of Population Health Sciences, Georgia State University School of Public Health

[b] Gangarosa Department of Environmental Health, Emory University Rollins School of Public Health

[c] Department of Behavioral, Social, and Health Education Sciences, Emory University Rollins School of Public Health

[d] Universidad Técnica Luis Vargas Torres de Esmeraldas

[e] Department of Epidemiology, University of Michigan School of Public Health

[f] Rutgers Global Health Institute and Department of Biostatistics and Epidemiology, Rutgers School of Public Health

[g] Department of Environmental and Occupational Health Sciences, University of Washington School of Public Health

[h] Hubert Department of Global Health, Emory University Rollins School of Public Health

S2 Table. Child sex-disaggregated demographic characteristics (*n*=297)

**Characteristics**	**Total**		**Male**		**Female**	
	n	(%)	*n*	(%)	*n*	(%)
**Number of participants**	297		144	(48.5)	153	(51.5)
**Maternal characteristics**						
Age (mean [std] in years)	29	(8.0)	30	(9.0)	29	(8.0)
Ethnicity						
Afro-Ecuadorian	221	(74.4)	108	(75.0)	113	(73.9)
Mestizo	70	(23.6)	32	(22.2)	38	(24.8)
Indigenous - Chachi	2	(0.7)	2	(1.4)	0	(0.0)
Manabí	3	(1.1)	1	(0.7)	2	(1.3)
Other	1	(0.4)	1	(0.7)	0	(0.0)
Education (mean [std] in years)	11.5	(3.5)	12	(3.5)	11	(3.5)
**Child characteristics**						
Age (mean [std] in months)	33	(15.5)	34	(15.0)	32	(16.0)
Currently breastfed	34	(11.4)	12	(8.3)	22	(14.4)
Symptoms in last 7 days						
Diarrhea	40	(13.5)	19	(13.2)	21	(13.7)
Fever	75	(25.3)	43	(29.9)	32	(20.9)
Vomit	23	(7.7)	10	(6.9)	13	(8.5)
Blood in stool	1	(0.3)	1	(0.7)	0	(0.0)
**Household characteristics**						
Number of people* (mean [std])	5	(2.5)	5.0	(2.0)	5	(2.0)
Owns animal(s)	165	(55.6)	82	(56.9)	83	(54.2)
Dogs	115	(38.7)	60	(41.7)	55	(35.9)
Cats	62	(20.9)	26	(18.1)	36	(23.5)
Creole chickens	35	(11.8)	18	(12.5)	17	(11.1)
Ducks	3	(1.0)	1	(0.7)	2	(1.3)
Dairy cattle	2	(0.7)	2	(1.4)	0	(0.0)
Horses	1	(0.3)	0	(0.0)	1	(0.7)
Pigs	12	(4.0)	6	(4.2)	6	(3.9)
Rabbits	7	(2.4)	3	(2.1)	4	(2.6)
Source of drinking water						
Piped	54	(18.2)	25	(17.4)	29	(19.0)
Bottled/purchased	170	(57.2)	85	(59.0)	85	(55.6)
Protected well	9	(3.0)	3	(2.1)	6	(3.9)
Rain water	43	(14.5)	20	(13.9)	23	(15.0)
Unprotected well	1	(0.3)	1	(0.7)	0	(0.0)
River water	4	(1.3)	2	(1.4)	2	(1.4)
Tanker-truck	16	(5.4)	9	(6.2)	7	(4.6)
Treat drinking water	88	(29.6)	43	(29.9)	45	(29.4)
Source(s) of water for child+						
Piped	108	(36.4)	59	(41.0)	49	(32.0)
Bottled/purchased	81	(27.3)	32	(23.6)	47	(30.7)
Protected well	26	(8.8)	11	(7.6)	15	(9.8)
Rain water	49	(16.5)	21	(14.6)	28	(18.3)
River water	11	(3.7)	5	(3.5)	6	(3.3)
Tanker-truck	32	(10.8)	18	(12.5)	14	(9.2)
Treat water for child	114	(38.4)	52	(36.1)	62	(40.5)

*n=292, Five observations have missing values

+Participants could report more than one source of water for their child so totals may add to more than 100%
