# Supplementary material for: The development and validation of a survey to measure fecal-oral child exposure to zoonotic enteropathogens: The FECEZ Enteropathogens Index
Source: PLOS Glob Public Health. 2024 Sep 10;4(9):e0002690. doi: 10.1371/journal.pgph.0002690 (PMC11386431; doi:10.1371/journal.pgph.0002690)
Supplement: S3 Table — (PDF) [file pgph.0002690.s008.pdf]

The development and validation of a survey to measure fecal-oral child exposure to zoonotic enteropathogens: The FECEZ Enteropathogens Index

S3 Table

April M. Ballard[a,b], Regine Haardörfer[c], Betty Corozo Angulo[d], Matthew C. Freeman[b], Joseph N.S. Eisenberg[e], Gwenyth O. Lee[f], Karen Levy[g], Bethany A. Caruso[h]

[a] Department of Population Health Sciences, Georgia State University School of Public Health

[b] Gangarosa Department of Environmental Health, Emory University Rollins School of Public Health

[c] Department of Behavioral, Social, and Health Education Sciences, Emory University Rollins School of Public Health

[d] Universidad Técnica Luis Vargas Torres de Esmeraldas

[e] Department of Epidemiology, University of Michigan School of Public Health

[f] Rutgers Global Health Institute and Department of Biostatistics and Epidemiology, Rutgers School of Public Health

[g] Department of Environmental and Occupational Health Sciences, University of Washington School of Public Health

[h] Hubert Department of Global Health, Emory University Rollins School of Public Health

S3 Table. Child exposure item frequencies (n=297)

Sub-domain and associated survey items	Frequency (%)					
	**Never**	**Rarely**	**Sometimes**	**Frequently**	**Frequency ratio**	**Percent unique data points**
***Child Environment (CE)* – Measure items**						
CE13. Dogs spend time outside near the house	32 (10.8)	5 (1.7)	17 (5.7)	243 (81.8)	7.6	1.3
CE14. Dogs enter the house	167 (56.2)	25 (8.4)	33 (11.1)	72 (24.2)	2.3	1.3
CE15. Dogs sleep inside the house	259 (87.2)	5 (1.7)	3 (1.0)	30 (10.1)	8.6	1.3
CE22. Dairy cattle spend time outside near the house	285 (96.0)	1 (0.3)	4 (1.3)	7 (2.4)	40.7	1.3
CE31. Cats spend time outside near the house	149 (50.2)	7 (2.4)	17 (5.7)	124 (41.8)	1.2	1.3
CE32. Cats enter the house	185 (62.3)	6 (2.0)	34 (11.4)	72 (24.2)	2.6	1.3
CE33. Cats sleep inside the house	249 (83.8)	2 (0.7)	9 (3.0)	37 (12.5)	6.7	1.3
CE34. Creole chickens spend time outside near the house	216 (72.7)	2 (0.7)	5 (1.7)	74 (24.9)	2.9	1.3
CE35. Creole chickens enter the house	267 (89.9)	6 (2.0)	8 (2.7)	16 (5.4)	16.7	1.3
CE54. Dog poop outside the house near or in the yard	117 (39.4)	13 (4.4)	42 (14.1)	125 (42.1)	1.1	1.3
CE58. Dairy cattle poop outside the house near or in the yard	294 (99.0)	1 (0.3)	2 (0.7)	0 (0.0)	147.0	1.0
CE66. Cat poop outside the house near or in the yard	284 (95.6)	1 (0.3)	5 (1.7)	7 (2.4)	40.6	1.3
CE68. Creole chicken poop outside the house near or in the yard	254 (85.5)	4 (1.3)	5 (1.7)	34 (11.4)	7.5	1.3
CE69. Creole chicken poop inside the house	289 (97.3)	2 (0.7)	2 (0.7)	4 (1.3)	72.3	1.3
CE90. House member (apart from mother and child under 5 years) work or care for an animal	263 (88.6)	19 (6.4)	9 (3.0)	6 (2.0)	13.8	1.3
CE92. Mother personally feeds or gives water to an animal	151 (50.8)	4 (1.3)	16 (5.4)	126 (42.4)	1.2	1.3
CE93. Mother personally touches or plays with an animal	196 (66.0)	11 (3.7)	16 (5.4)	74 (24.9)	2.6	1.3
CE94. Mother personally bathes, cleans, or grooms an animal	241 (81.1)	4 (1.3)	17 (5.7)	35 (11.8)	6.9	1.3
CE95. Mother personally cleans the habitat or place where an animal sleeps and/or defecates	210 (70.7)	7 (2.4)	17 (5.7)	63 (21.2)	3.3	1.3
CE97. Mother personally eliminates or cleans the poop of an animal	203 (68.4)	3 (1.0)	24 (8.1)	67 (22.6)	3.0	1.4
***Child Behavior (CB)* – Measure items**						
CB80. Child plays with or carries around shoes like a toy	73 (24.6)	13 (4.4)	39 (13.1)	172 (57.9)	2.4	1.3
CB81. Child plays in soil or dirt outside the house	54 (18.2)	27 (9.1)	35 (11.8)	181 (60.9)	3.4	1.3

Sub-domain and associated survey items	Frequency (%) Never	Rarely	Sometimes	Frequently	Frequency ratio	Percent unique data points
Child Behavior (CB) **– Measure items**						
CB83. Child plays outside the house in an area where an animal lives or sleeps	100 (33.7)	29 (9.8)	35 (11.8)	133 (44.8)	1.3	1.3
CB84. Child plays outside the house without shoes on	119 (40.1)	15 (5.1)	43 (14.5)	120 (40.4)	1.0	1.3
CB85. Child puts objects or toys that had contact with the floor inside the house in their mouth	120 (40.4)	22 (7.4)	38 (12.8)	117 (39.4)	1.0	1.3
CB86. Child puts objects or toys that had contact with the dirt outside the house in their mouth	215 (72.4)	7 (2.4)	18 (6.1)	57 (19.2)	3.8	1.3
CB87. Child puts dirt or soil in their mouth	243 (81.8)	10 (3.4)	8 (2.7)	36 (12.1)	6.8	1.3
CB89. Child puts shoes in their mouth	217 (73.1)	10 (3.4)	24 (8.1)	46 (15.5)	4.7	1.3
CB98. Child feeds or gives water or helps others feed or give water to an animal	253 (85.2)	9 (3.0)	11 (3.7)	24 (8.1)	10.5	1.3
CB99. Child touches or plays with an animal	159 (53.5)	20 (6.7)	25 (8.4)	93 (31.3)	1.7	1.3
CB100. Child bathes, cleans, or grooms or helps others bathe, clean, or groom an animal	282 (94.9)	2 (0.7)	4 (1.3)	9 (3.0)	31.3	1.3
CB101. Child cleans or helps others clean the habitat or place where an animal sleeps and/or defecates	289 (97.3)	1 (0.3)	3 (1.0)	4 (1.3)	72.3	1.3
CB102. Child cares for or helps others care for an animal that was sick	294 (99.0)	1 (0.3)	2 (0.7)	0 (0.0)	147.0	1.0
CB104. Child touches, removes, or cleans animal poop	293 (98.7)	1 (0.3)	2 (0.7)	1 (0.3)	146.5	1.3
Child Environment (CE) **– Deleted items**						
CE1. Production chickens spend time outside near the house	297 (100.0)	0 (0.0)	0 (0.0)	0 (0.0)	0.0	0.3
CE2. Production chickens enter the house	297 (100.0)	0 (0.0)	0 (0.0)	0 (0.0)	0.0	0.3
CE3. Production chickens sleep inside the house	297 (100.0)	0 (0.0)	0 (0.0)	0 (0.0)	0.0	0.3
CE4. Ducks spend time outside near the house	292 (98.3)	1 (0.3)	1 (0.3)	3 (1.0)	97.3	1.3
CE5. Ducks enter the house	295 (99.3)	1 (0.3)	1 (0.3)	0 (0.0)	295.0	1.0
CE6. Ducks sleep inside the house	297 (100.0)	0 (0.0)	0 (0.0)	0 (0.0)	0.0	0.3
CE7. Turkeys spend time outside near the house	297 (100.0)	0 (0.0)	0 (0.0)	0 (0.0)	0.0	0.3
CE8. Turkeys enter the house	297 (100.0)	0 (0.0)	0 (0.0)	0 (0.0)	0.0	0.3

	Frequency (%)					
**Sub-domain and associated survey items**	**Never**	**Rarely**	**Sometimes**	**Frequently**	**Frequency ratio**	**Percent unique data points**
Child Environment (CE) **– Deleted items**						
CE9. Turkeys sleep inside the house	297 (100.0)	0 (0.0)	0 (0.0)	0 (0.0)	0.0	0.3
CE10. Guinea pigs spend time outside near the house	297 (100.0)	0 (0.0)	0 (0.0)	0 (0.0)	0.0	0.3
CE11. Guinea pigs enter the house	297 (100.0)	0 (0.0)	0 (0.0)	0 (0.0)	0.0	0.3
CE12. Guinea pigs sleep inside the house	297 (100.0)	0 (0.0)	0 (0.0)	0 (0.0)	0.0	0.3
CE16. Pigs spend time outside near the house	283 (95.3)	0 (0.0)	1 (0.3)	13 (4.4)	21.8	1.0
CE17. Pigs enter the house	296 (99.7)	0 (0.0)	0 (0.0)	1 (0.3)	296.0	0.7
CE18. Pigs sleep inside the house	296 (99.7)	0 (0.0)	0 (0.0)	1 (0.3)	296.0	0.7
CE19. Cattle spend time outside near the house	297 (100.0)	0 (0.0)	0 (0.0)	0 (0.0)	0.0	0.3
CE20. Cattle enter the house	297 (100.0)	0 (0.0)	0 (0.0)	0 (0.0)	0.0	0.3
CE21. Cattle sleep inside the house	297 (100.0)	0 (0.0)	0 (0.0)	0 (0.0)	0.0	0.3
CE23. Dairy cattle enter the house	297 (100.0)	0 (0.0)	0 (0.0)	0 (0.0)	0.0	0.3
CE24. Dairy cattle sleep in the house	297 (100.0)	0 (0.0)	0 (0.0)	0 (0.0)	0.0	0.3
CE25. Horses/mules/donkeys spend time outside near the house	294 (99.0)	0 (0.0)	0 (0.0)	3 (1.0)	98.0	0.7
CE26. Horses/mules/donkeys enter the house	297 (100.0)	0 (0.0)	0 (0.0)	0 (0.0)	0.0	0.3
CE27. Horses/mules/donkeys sleep inside the house	297 (100.0)	0 (0.0)	0 (0.0)	0 (0.0)	0.0	0.3
CE28. Sheep/goats spend time outside near the house	296 (99.7)	0 (0.0)	1 (0.3)	0 (0.0)	296.0	0.7
CE29. Sheep/goats enter the house	296 (99.7)	0 (0.0)	1 (0.3)	0 (0.0)	296.0	0.7
CE30. Sheep/goats sleep inside the house	296 (99.7)	0 (0.0)	1 (0.3)	0 (0.0)	296.0	0.7
CE34. Creole chickens sleep inside the house	290 (97.6)	1 (0.3)	1 (0.3)	5 (1.7)	58.0	1.3
CE37. Bushrats spend time outside near the house	297 (100.0)	0 (0.0)	0 (0.0)	0 (0.0)	0.0	0.3
CE38. Bushrats enter the house	297 (100.0)	0 (0.0)	0 (0.0)	0 (0.0)	0.0	0.3
CE39. Bushrats sleep inside the house	297 (100.0)	0 (0.0)	0 (0.0)	0 (0.0)	0.0	0.3
CE40. Rats/rodents spend time outside near the house	289 (97.3)	4 (1.3)	4 (1.3)	0 (0.0)	72.3	1.0
CE41. Rats/rodents enter the house	289 (97.3)	1 (0.3)	5 (1.7)	2 (0.7)	57.8	1.3
CE42. Rats/rodents sleep inside the house	289 (97.3)	0 (0.0)	6 (2.0)	2 (0.7)	48.2	1.0
CE43. Rabbits spend time outside near the house	292 (98.3)	1 (0.3)	0 (0.0)	4 (1.3)	73.0	1.0

	Frequency (%)					
**Sub-domain and associated survey items**	**Never**	**Rarely**	**Sometimes**	**Frequently**	**Frequency ratio**	**Percent unique data points**
***Child Environment (CE)* – Deleted items**						
CE44. Rabbits enter the house	291 (98.0)	0 (0.0)	0 (0.0)	6 (2.0)	48.5	0.7
CE45. Rabbits sleep inside the house	291 (98.0)	0 (0.0)	0 (0.0)	6 (2.0)	48.5	0.7
CE46. Production chicken poop outside the house near or in the yard	297 (100.0)	0 (0.0)	0 (0.0)	0 (0.0)	0.0	0.3
CE47. Production chicken poop inside the house	297 (100.0)	0 (0.0)	0 (0.0)	0 (0.0)	0.0	0.3
CE48. Duck poop outside the house near or in the yard	296 (99.7)	0 (0.0)	0 (0.0)	1 (0.3)	296.0	0.7
CE49. Duck poop inside the house	296 (99.7)	1 (0.3)	0 (0.0)	0 (0.0)	296.0	0.7
CE50. Turkey poop outside the house near or in the yard	297 (100.0)	0 (0.0)	0 (0.0)	0 (0.0)	0.0	0.3
CE51. Turkey poop inside the house	297 (100.0)	0 (0.0)	0 (0.0)	0 (0.0)	0.0	0.36
CE52. Guinea pig poop outside the house near or in the yard	297 (100.0)	0 (0.0)	0 (0.0)	0 (0.0)	0.0	0.3
CE53. Guinea pig poop inside the house	297 (100.0)	0 (0.0)	0 (0.0)	0 (0.0)	0.0	0.36
CE55. Dog poop inside the house	283 (95.3)	2 (0.7)	5 (1.7)	7 (2.4)	40.4	1.3
CE56. Pig poop outside the house near or in the yard	295 (99.3)	0 (0.0)	1 (0.3)	1 (0.3)	295.0	1.0
CE57. Pig poop inside the house	297 (100.0)	0 (0.0)	0 (0.0)	0 (0.0)	0.0	0.3
CE58. Cattle poop outside the house near or in the yard	297 (100.0)	0 (0.0)	0 (0.0)	0 (0.0)	0.0	0.3
CE59. Cattle poop inside the house	297 (100.0)	0 (0.0)	0 (0.0)	0 (0.0)	0.0	0.3
CE61. Dairy cattle poop inside the house	297 (100.0)	0 (0.0)	0 (0.0)	0 (0.0)	0.0	0.36
CE62. Horses/mule/donkey poop outside the house near or in the yard	293 (98.7)	0 (0.0)	0 (0.0)	4 (1.3)	73.3	0.7
CE63. Horses/mule/donkey poop inside the house	297 (100.0)	0 (0.0)	0 (0.0)	0 (0.0)	0.0	0.3
CE64. Sheep/goat poop outside the house near or in the yard	297 (100.0)	0 (0.0)	0 (0.0)	0 (0.0)	0.0	0.3
CE65. Sheep/goat poop inside the house	297 (100.0)	0 (0.0)	0 (0.0)	0 (0.0)	0.0	0.3
CE67. Cat poop inside the house	293 (98.7)	1 (0.3)	0 (0.0)	3 (1.0)	97.7	1.0
CE70. Bushrat poop outside the house near or in the yard	297 (100.0)	0 (0.0)	0 (0.0)	0 (0.0)	0.0	0.3
CE71. Bushrat poop inside the house	297 (100.0)	0 (0.0)	0 (0.0)	0 (0.0)	0.0	0.3
CE72. Rat/rodent poop outside the house near or in the yard	293 (98.7)	1 (0.3)	1 (0.3)	2 (0.7)	146.5	1.3
CE73. Rat/rodent poop inside the house	293 (98.7)	0 (0.0)	3 (1.0)	1 (0.3)	97.7	1.0

	Frequency (%)					
**Sub-domain and associated survey items**	**Never**	**Rarely**	**Sometimes**	**Frequently**	**Frequency ratio**	**Percent unique data points**
***Child Environment (CE)* – Deleted items**						
CE74. Rabbit poop outside the house near or in the yard	296 (99.7)	0 (0.0)	0 (0.0)	1 (0.3)	296.0	0.7
CE75. Rabbit poop inside the house	296 (99.7)	0 (0.0)	1 (0.3)	0 (0.0)	296.0	0.7
CE76. Poop from an unknown type of animal outside the house near or in the yard	297 (100.0)	0 (0.0)	0 (0.0)	0 (0.0)	0.0	0.3
CE77. Poop from an unknown type of animal inside the house	297 (100.0)	0 (0.0)	0 (0.0)	0 (0.0)	0.0	0.3
CE91. Mother or someone who lives in the same house puts or throws leftover food outside for an animal	175 (58.9)	17 (5.7)	29 (9.8)	76 (25.6)	2.3	1.3
CE96. Mother personally cares for an animal that was sick	285 (96.0)	3 (1.0)	1 (0.3)	8 (2.7)	35.6	1.3
***Child Behavior (CB)* – Deleted items**						
CB78. Child plays on the floor of the house without a rug or playmat	25 (8.4)	6 (2.0)	18 (6.1)	248 (83.5)	9.9	1.3
CB79. Child plays inside the house in an area where an animal spends time or sleeps	169 (56.9)	16 (5.4)	25 (.84)	87 (29.3)	1.9	1.3
CB82. Child plays in sand outside the house	60 (20.2)	27 (9.1)	35 (11.8)	175 (58.9)	2.9	1.3
CB88. Child puts sand in their mouth	244 (82.2)	11 (3.7)	7 (2.4)	35 (11.8)	7.0	1.3
CB103. Child touches or plays with objects used to remove or clean animal poop such as brooms or shovels	272 (91.6)	3 (1.0)	12 (4.0)	10 (3.4)	22.7	1.3
CB105. Child puts animal poop in their mouth	297 (100.0)	0 (0.0)	0 (0.0)	0 (0.0)	0.0	0.3
